# Identification of clinical disease trajectories in neurodegenerative disorders with natural language processing

**DOI:** 10.1038/s41591-024-02843-9

**Published:** 2024-03-12

**Authors:** Nienke J. Mekkes, Minke Groot, Eric Hoekstra, Alyse de Boer, Ekaterina Dagkesamanskaia, Sander Bouwman, Sophie M. T. Wehrens, Megan K. Herbert, Dennis D. Wever, Annemieke Rozemuller, Bart J. L. Eggen, Inge Huitinga, Inge R. Holtman

**Affiliations:** 1 Department of Biomedical Sciences, Section Molecular Neurobiology, University of Groningen, University Medical Center Groningen, Groningen, The Netherlands; 2 Machine Learning Lab, Data Science Center in Health, University of Groningen, University Medical Center Groningen, Groningen, The Netherlands; 3 The Netherlands Brain Bank, Netherlands Institute for Neuroscience, Amsterdam, The Netherlands; 4 Department of Pathology, Amsterdam UMC loc. VUmc, Amsterdam, The Netherlands; 5 Swammerdam Institute for Life Sciences, University of Amsterdam, Amsterdam, The Netherlands

**Keywords:** Neurodegenerative diseases, Dementia, Signs and symptoms, Data integration, Machine learning

## Abstract

Neurodegenerative disorders exhibit considerable clinical heterogeneity and are frequently misdiagnosed. This heterogeneity is often neglected and difficult to study. Therefore, innovative data-driven approaches utilizing substantial autopsy cohorts are needed to address this complexity and improve diagnosis, prognosis and fundamental research. We present clinical disease trajectories from 3,042 Netherlands Brain Bank donors, encompassing 84 neuropsychiatric signs and symptoms identified through natural language processing. This unique resource provides valuable new insights into neurodegenerative disorder symptomatology. To illustrate, we identified signs and symptoms that differed between frequently misdiagnosed disorders. In addition, we performed predictive modeling and identified clinical subtypes of various brain disorders, indicative of neural substructures being differently affected. Finally, integrating clinical diagnosis information revealed a substantial proportion of inaccurately diagnosed donors that masquerade as another disorder. The unique datasets allow researchers to study the clinical manifestation of signs and symptoms across neurodegenerative disorders, and identify associated molecular and cellular features.

## Abstract (web summary)

Analyzing clinical records from a cohort of 3,042 donors in the Netherlands Brain Bank, natural language processing models unveil the symptomatology and potential misdiagnoses of a broad range of neurodegenerative disorders.

## Main

The brain is a highly complex organ that is susceptible to a wide range of neurodegenerative disorders that can result in dementia, including Alzheimer’s disease (AD), subtypes of frontotemporal dementia (FTD), Parkinson’s disease (PD), dementia with Lewy bodies (DLB), vascular dementia (VD) and mixed forms of dementia. The incidence of dementia is expected to triple by 2050 (ref. ^1^) and is the seventh leading cause of death worldwide with tremendous economic impact. Importantly, the number of treatment options for these disorders is still very limited and more fundamental research is crucial^2^. Most dementias are difficult to diagnose and study due to considerable heterogeneity^3–5^, partially shared clinical and pathological features^6,7^ and complex comorbidity patterns^8,9^. The relationship between neuropathological diagnosis (ND) and clinical manifestation is complex, with partially overlapping signs and symptoms manifesting in various disorders. This frequently results in discrepancies between clinical and postmortem ND, with up to a third of cases with a specific dementia being clinically misdiagnosed^10,11^. However, the frequency and the temporal profiles of these signs and symptoms generally tend to differ. Hence, it is crucially important to establish new global approaches that aim to systematically obtain and harmonize clinical and neuropathological information.

Brain banks that disseminate postmortem brain tissues have fueled worldwide research into neurodegenerative diseases and, together with molecular biology and biochemical assays, genomics technologies and microscopic imaging, have given unprecedented insight into underlying pathophysiological mechanisms. However, a major limitation of current postmortem dementia studies is that most brain banks collect and supply very limited clinical information, hampering the ability to include key clinical parameters in the statistical designs of postmortem studies. Many brain studies continue to use a binary case–control design, overlooking the phenotypic diversity among cases and controls. Although there have been attempts to integrate clinical diagnosis (CD), clinical symptoms or temporal profiling, to the best of our knowledge, these approaches have not been comprehensively combined. To address this issue, we aimed to delineate clinical disease trajectories across neuropathologically defined brain disorders by mining the medical record summaries from donors of the Netherlands Brain Bank (NBB).

The NBB is a nonprofit organization that currently has performed over 5,000 human brain autopsies^12^ and is renowned for brain tissue with short postmortem delay and extensive medical record summaries. This makes the NBB a highly valuable resource that has facilitated neuroscientific research globally. However, these unstructured medical record summaries had not yet been converted into a standardized format necessary for scientific purposes. To convert these medical record summaries into clinical disease trajectories, we developed a computational pipeline consisting of parsers and natural language processing (NLP) techniques. These clinical disease trajectories can be used to facilitate fundamental research questions, such as the identification of clinical subtypes and the investigation of heterogeneity within disorders, and could contribute toward a more individualized medicine approach.

By integrating these clinical disease trajectories with the neuropathologically defined diagnosis, we were able to perform temporal profiling and survival analysis of various brain disorders. We also compared the accuracy of the CDs with that of the NDs assigned by the neuropathologist, seen as the ground truth. Finally, we illustrate how this dataset can be used for the predictive modeling of brain disorders and the identification of new data-driven clinical subtypes of disease, including subtypes of dementia, subtypes of early and late PD and subtypes of multiple sclerosis (MS).

## Results

### Identification of neuropsychiatric signs and symptoms and exploration of the labeled data

**Fig. 1:**
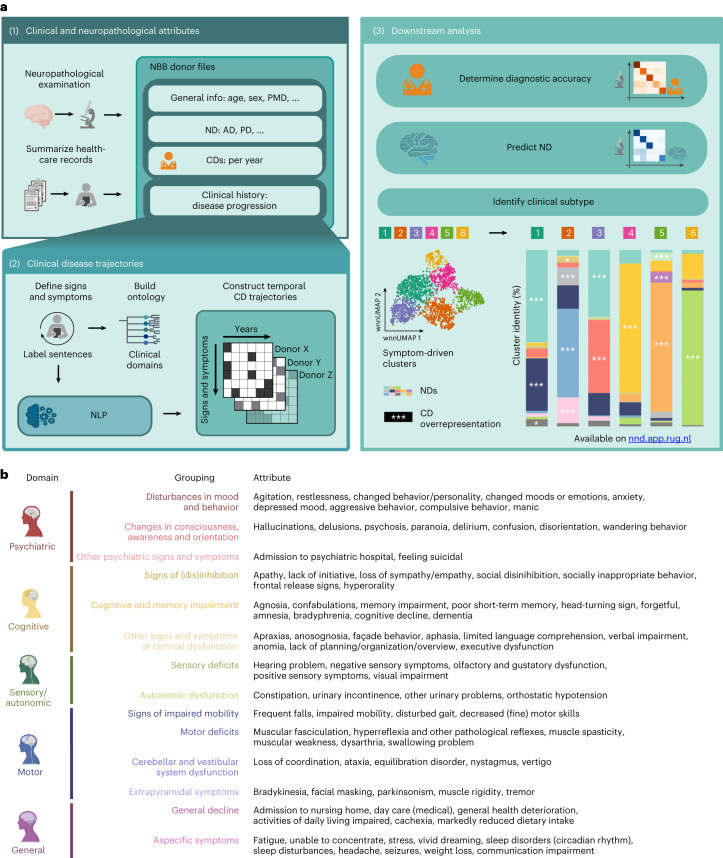
Introduction to the project. **a**, Workflow of the project describing the different data types in the NBB donor files (i), the processing of the clinical history data resulting in clinical disease trajectories (ii) and downstream analyses (iii). **b**, Clinical attributes (signs and symptoms), their domains, and groupings, including colors and illustrative brain icons. Relevant data, meta-data and analyses for this project can be found on https://nnd.app.rug.nl.

**Extended Data Fig. 1:**
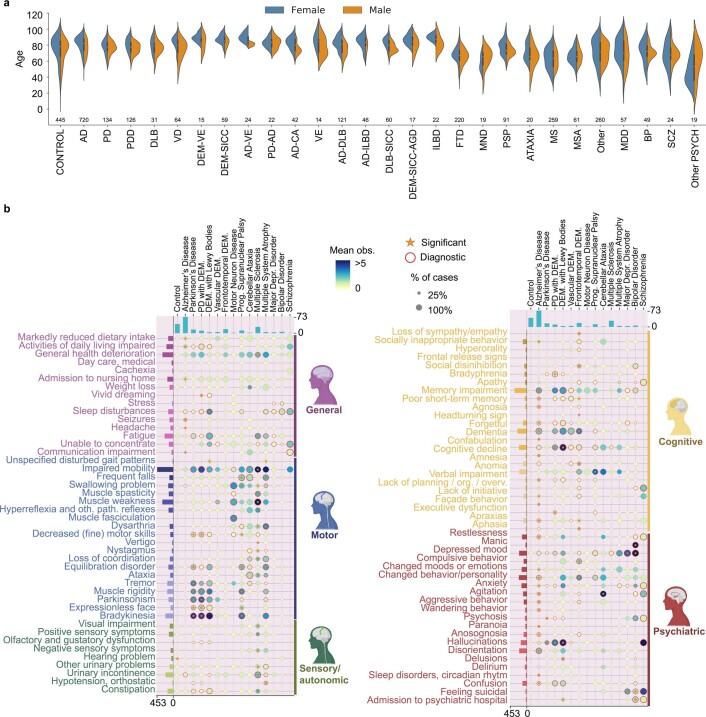
Dataset characteristics and training data attribute distribution. General outline of all data. **a**) Violin plot (center marker, box limits and whiskers represent the median, interquartile range and 1.5× interquartile range) showing the age and number of donors (n = 3,042 total individual donors) of the most common Neuropathological Diagnoses, separated by sex. **b**) Integrated dot and bar plot showing the manifestation of signs and symptoms (Y-axis) by diagnoses (X-axis) for the labeled dataset. The dot size corresponds to the proportion of donors in which a sign/symptoms was observed. The dot color corresponds to the mean number of observations of a sign/symptom across donors. Signs and symptoms important for diagnostics are highlighted in orange. Significantly overrepresented signs/symptoms were visualized with an asterisk (one-sided permutation test, FDR corrected P < 0.1).

We have established a computational pipeline that consists of text parsers and NLP models to convert the extensive medical record summaries into clinical disease trajectories (Fig. 1a). This pipeline consists of three steps, with the first parsing NBB donor files, the second defining and predicting attributes in the clinical history (Extended Data Table 1) and converting the predicted signs and symptoms into clinical disease trajectories, and the third using the trajectories for downstream analyses. In total, we included 3,042 donor files from donors with various NDs (Extended Data Fig. 1a, Table 1 and Supplementary Tables 1 and 2).

**Table 1:** Overview of the most common NDs and corresponding abbreviations, including ICD-10 codes

Full name	Abbreviation	Type	ICD-10	*n*
Alzheimer’s disease	AD	Progressive neurodegenerative disease	G30	720
Cerebellar ataxia	ATAXIA	Progressive neurodegenerative disease	G11	20
Bipolar disorder	BP	Psychiatric disorder	F31	49
Control donor	CON	Control donor (without a clinical or neuropathological indication of brain disorder)		445
Dementia with Lewy bodies	DLB	Progressive neurodegenerative disease	G31.8	31
Frontotemporal dementia	FTD	Progressive neurodegenerative disease	G31.0	220
Major depressive disorder	MDD	Psychiatric disorder	F32	57
Motor neuron disease	MND	Progressive neurodegenerative disease	G12.2	19
Multiple sclerosis	MS	Neuroinflammatory disease	G35	259
Multiple system atrophy	MSA	Progressive neurodegenerative disease	G23.2 G23.3	61
Parkinson’s disease	PD	Progressive neurodegenerative disease	G20	134
Parkinson’s disease with dementia	PDD	Progressive neurodegenerative disease	G20	126
Progressive supranuclear palsy	PSP	Progressive neurodegenerative disease		91
Schizophrenia	SCZ	Psychiatric disorder	F20	24
Vascular dementia	VD	Vascular disorder	F01	64

First, we defined a new crossdisorder clinical categorization system that contains 90 neuropsychiatric signs and symptoms, associated with brain disorders and overall wellbeing/functioning, across 5 broad domains (Fig. 1b). From a random set of 293 donors, 18,917 sentences were scored by 1 scorer to create a dataset to refine, validate and test different NLP models (Supplementary Table 3). To determine the reliability of the scoring process, 1,000 sentences were randomly selected and scored independently by another scorer. The interannotator agreement was high, corroborating the reliability of our gold standard (Cohen’s *κ* = 0.86). Next, we performed an enrichment analysis to determine whether the labeled signs and symptoms were more frequently observed in each disorder than expected by random chance. This analysis identified many expected disease-specific signs and symptoms such as ‘dementia’ being significantly enriched in AD, PDD, DLB and VD but not in PD without dementia and ‘bradykinesia’ in PD, PDD, MSA and PSP, disorders that are known to exhibit extrapyramidal symptoms (Extended Data Fig. 1b). These observed neuropsychiatric signs and symptoms were significantly overrepresented for a priori defined signs and symptoms of diagnostic importance (*χ*^2^ = 171.28, *P* = 1 × 10^−31^).

### Refining NLP models and constructing clinical disease trajectories

**Extended Data Fig. 2:**
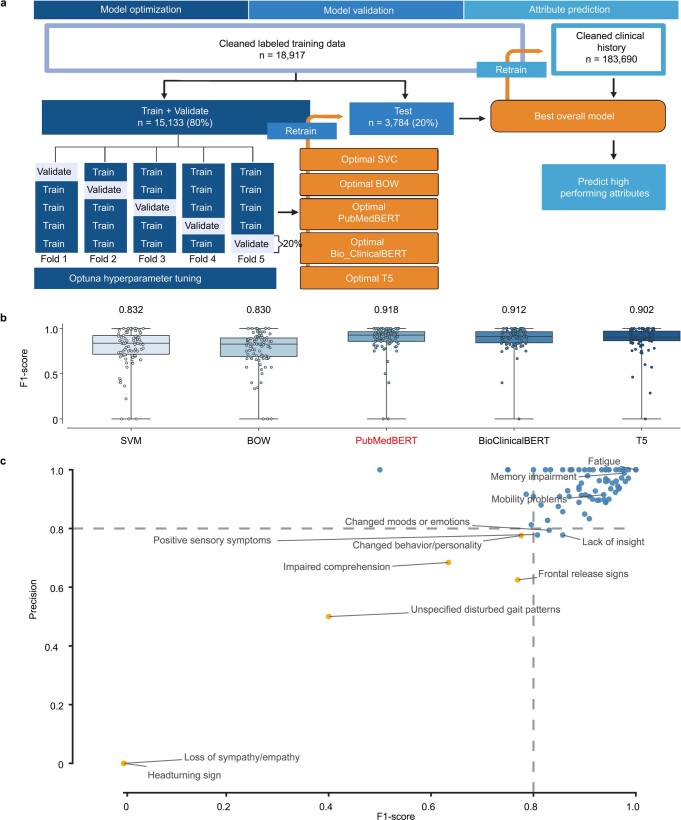
NLP workflow, comparison, and individual attribute performance. Natural Language Processing. **a**) NLP workflow. The workflow consists of three main phases: model optimization, model validation, and attribute prediction. **b**) Boxplots (center line, box limits and whiskers represent the median, interquartile range and 1.5× interquartile range) showing the micro F1-score per sign or symptom (n = 90 total signs or symptoms) on the test data. The best performing model, PubMedBERT, was highlighted in red. **c**) Scatterplot depicting the classification performance of PubMedBERT in the form of precision and F1-score for individual signs and symptoms on the test data.

To reliably identify neuropsychiatric signs and symptoms in individual sentences, we established a pipeline to refine and compare different NLP model architectures (Extended Data Fig. 2a). The data were divided into a training and a hold-out test set, stratified according to a relatively equal distribution of sign and symptom observations. We then employed a stratified fivefold crossvalidation approach, where models were refined in fourfold and validated on the remaining part of the data. Five different model architectures (bag of words model (BOW), support vector machine (SVM), Bio_ClinicalBERT, PubMedBERT and T5) were refined and optimized with Optuna, and the best performing model, according to average micro-*F*1-score and average micro-precision, was selected. Almost all signs and symptoms were reliably identified by all models, but a small subset of six signs and symptoms performed considerably less well. These consistently included the same attributes and were subsequently excluded. Next, the highest scoring iterations of each model architecture were compared using the hold-out test data, on which PubMedBERT showed the best model performance (Extended Data Fig. 2b). The optimal PubMedBERT architecture was fine-tuned again on all labeled data for the prediction of the 84 remaining signs and symptoms that exhibited a micro-precision ≥0.8 or a micro-*F*1-score ≥0.8 (Extended Data Fig. 2c). This final model was then used to predict whether specific signs or symptoms were described in individual sentences of the full corpus. To construct the final clinical disease trajectories (Supplementary Table 4), the predictions of multiple sentences were collapsed per year. These new clinical disease trajectories encompass a wider range of neuropsychiatric signs and symptoms, covering a longer time frame, and include a larger number of donors compared with what has been previously published (Supplementary Table 5).

### Interpretation of signs and symptoms across common brain disorders

**Fig. 2:**
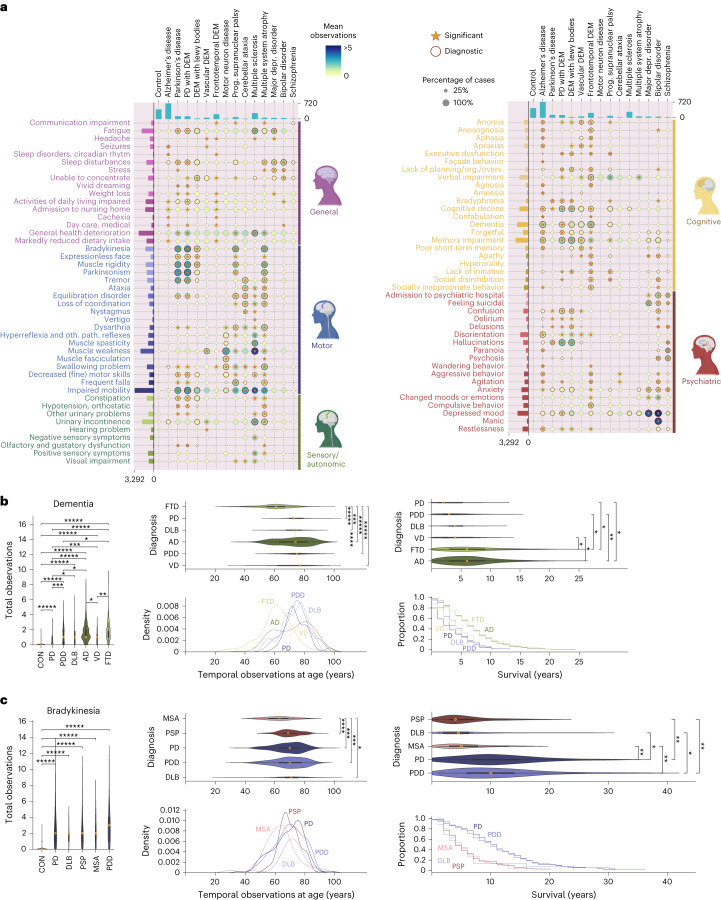
Clinical disease trajectories offer a wealth of information. **a**, Integrated plot showing attribute (*y* axis) manifestation by NDs (*x* axis). The dot size corresponds to the proportion of donors in which an attribute was observed. The dot color corresponds to the mean number of observations of an attribute across donors. Orange highlight and asterisks represent attributes important for diagnostics and significantly overrepresented signs/symptoms (one-sided permutation test, FDR-corrected *P* < 0.1), respectively. oth. path., other pathological. **b**, ‘Dementia’ temporal profiling (*n* = 1,326 donors, of which *n* = 682 with ≥1 ‘dementia’) showing density plot, Kaplan–Meier plot and three violin plots (center marker, box limits and whiskers represent the median, interquartile range (IQR) and 1.5× IQR). Two-sided Mann–Whitney *U*-test, FDR-corrected *P* values: ^*^1.00 × 10^−4^ < *P* ≤ 1.00 × 10^−2^; ^**^1.00 × 10^−6^ < *P* ≤ 1.00 × 10^−4^; ^***^1.00 × 10^−8^ < *P* ≤ 1.00 × 10^−6^; ^****^1.00 × 10^−10^ < *P* ≤ 1.00 × 10^−8^; ^****^P ≤ 1.00 × 10^−10^. **c**, ‘Bradykinesia’ temporal profiling plots (*n* = 762 donors, of which *n* = 268 with ≥1 ‘bradykinesia’). All plots as defined in **b**.

The clinical disease trajectories represent a distinctive dataset documenting neuropsychiatric signs and symptoms observed on a yearly basis for each donor. Again, we performed an enrichment analysis to determine whether the predicted signs or symptoms were more frequently observed in each disorder than expected (Fig. 2a). Of the signs and symptoms, 269 were significantly enriched in specific diagnoses, of which 148 were also a priori defined to be of diagnostic importance, a highly significant enrichment *(χ*^2^ = 295.96, *P* = 2.5 × 10^−66^). Importantly, the enrichment of the predicted dataset for a priori predicted signs and symptoms is much more pronounced than the labeled dataset, offering orthogonal evidence for the validity of our NLP approach.

It is interesting that all neuropsychiatric signs and symptoms were significantly enriched in at least one brain disorder, suggesting that all these signs and symptoms were indeed relevant for (a subset) of disorders. As expected, ‘dementia’ and ‘memory impairment’ were significantly enriched in dementias including AD, FTD, DLB, VD and PDD, but not in PD without dementia. Similarly, MS showed a striking enrichment for ‘impaired mobility’ and ‘muscle weakness’ and ‘fatigue’, which is very much in line with the disabling pathology of the brain and spinal cord. However, where ‘impaired mobility’ was significantly enriched in MS, PD, PDD, PSP, ATAXIA and MSA, ‘muscle weakness’ was enriched in VD, MND, PSP, MSA and MS, showing that our approach can detect a unique compendium of signs and symptoms in a disorder-specific manner.

Dementias are frequently clinically misdiagnosed. Hence, we aimed to determine whether we could identify neuropsychiatric signs and symptoms that could contribute to improved differential diagnosis between subsets of frequently misdiagnosed disorders. We found a number of signs and symptoms that were uniquely enriched in specific dementia subtypes, including ‘paranoia’, and ‘façade behavior’ in AD and ‘hearing problem’ and ‘muscle weakness’ in VD (Extended Data Table 2). Similarly, MSA, PD, PSP and DLB are frequently misdiagnosed^13,14^. We found that ‘depressed mood’ was unique to PDD, ‘apraxias’ in DLB, ‘ataxia’ and ‘muscle fasciculation’ in MSA and ‘visual impairment’ in PSP (Extended Data Table 3). These findings suggest that we retrospectively have created a unique dataset that describes the clinical signs and symptoms that are associated with various brain disorders, which could contribute to improved diagnosis.

### Temporal profiling of signs and symptoms across brain disorders

We utilized the clinical disease trajectories to conduct temporal profiling of specific neuropsychiatric signs and symptoms across various disorders. To this end, we calculated three different statistics. First, we calculated the total number of year observations in each condition in relation to the donors, to determine whether specific signs and symptoms were significantly more frequently observed in different diagnoses. Second, we calculated the temporal profile of those signs and symptoms, as a distribution of the years in which they were observed. Third, we performed a survival analysis to determine whether there are differences in the overall survival rate after the first observation of a sign or symptom between donors with different NDs. As expected, we observed that the attribute ‘dementia’ was present at a significantly younger age in FTD^15^ than in other dementias (Fig. 2b and Supplementary Table 6). The survival analysis showed that, after the first observation of ‘dementia’, the survival of donors with VD, PD or PDD was significantly shorter than donors with AD or FTD. These observations are in line with clinical expectations and corroborate the temporal validity of these clinical disease trajectories.

Synucleinopathies are neurological conditions that are characterized by α-synuclein protein aggregation, including PD, PDD, DLB and MSA. There is debate about whether these synucleinopathies are different manifestations of the same underlying neuropathology manifesting in different brain regions or whether there are unique neuropathological processes associated with each disorder^14,16^. By studying the temporal and survival profiles after the manifestation of specific symptoms, we can determine whether these disorders exhibit unique temporal features, suggesting qualitatively different neuropathological processes. To study this in more detail, we performed temporal profiling analyses with ‘bradykinesia’ (Fig. 2c and Supplementary Table 6). Similar to ‘dementia’ in FTD, we found that ‘bradykinesia’ was observed at a significantly younger age in MSA than in the other disorders. To the contrary, the survival analysis showed that donors with MSA, PSP and DLB with ‘bradykinesia’ had significantly shorter survival than donors with PD and PDD. These findings are in line with the hypothesis that there are qualitatively different aspects to these synucleinopathies, in which PD and PDD are very similar, but that DLB, and especially MSA, are uniquely different^14,16^. Both analyses corroborate the notion that many brain disorders exhibit partially overlapping clinical symptoms that manifest in a distinct temporal fashion, potentially indicative of the neuronal substructures that are affected.

**Extended Data Fig. 3:**
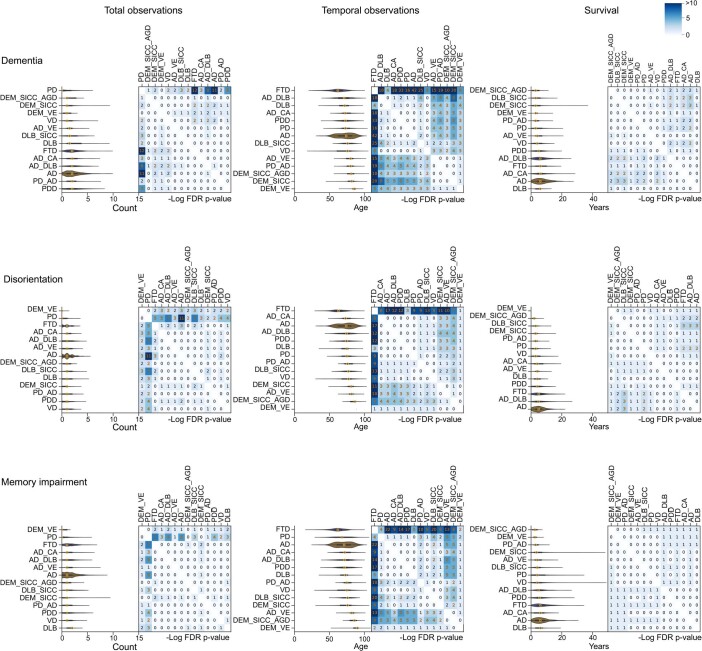
Observational and temporal analysis of rare and mixed dementias. Violin plots (center marker, box limits and whiskers represent the median, interquartile range and 1.5× interquartile range) depicting the observation distributions, the temporal distributions, and survival distributions for dementias with different causes (n = 1164 donors), including rare and mixed dementias, using ‘Dementia’ (n = 894 donors),’Disorientation’ (n = 722 donors) and ‘Memory Impairment’ (n = 860 donors). Each violin plot is accompanied by a heatmap showing the results of pairwise significance testing (two-sided Mann-Whitney U test), with −10log FDR corrected p-values depicted in orange when significant (p <= 0.01).

We next compared rare and mixed dementias, including dementia-vascular encephalopathy (DEM-VE), DEM with senile involutive cortical changes (DEM-SICC) and AD-VE. Dementias are a broad category of disorders and mixed and rare forms of dementia are frequently disregarded. We found that ‘dementia’ was observed at a significantly later age in several mixed forms of dementia, including AD-VE and AD-PD, than in AD and VD (Extended Data Fig. 3), suggesting that the pathogenesis generally strikes at later age in patients with these mixed disorders. Furthermore, survival analysis suggests that AD, DLB and FTD might exhibit an extended survival period after the manifestation of ‘dementia’ compared with several other subtypes of dementia. Our analysis deviates in certain aspects from previous studies^17,18^, in which the diagnosis was based only on clinical data. Future studies using neuropathologically defined cohorts are necessary to address these differences.

**Extended Data Fig. 4:**
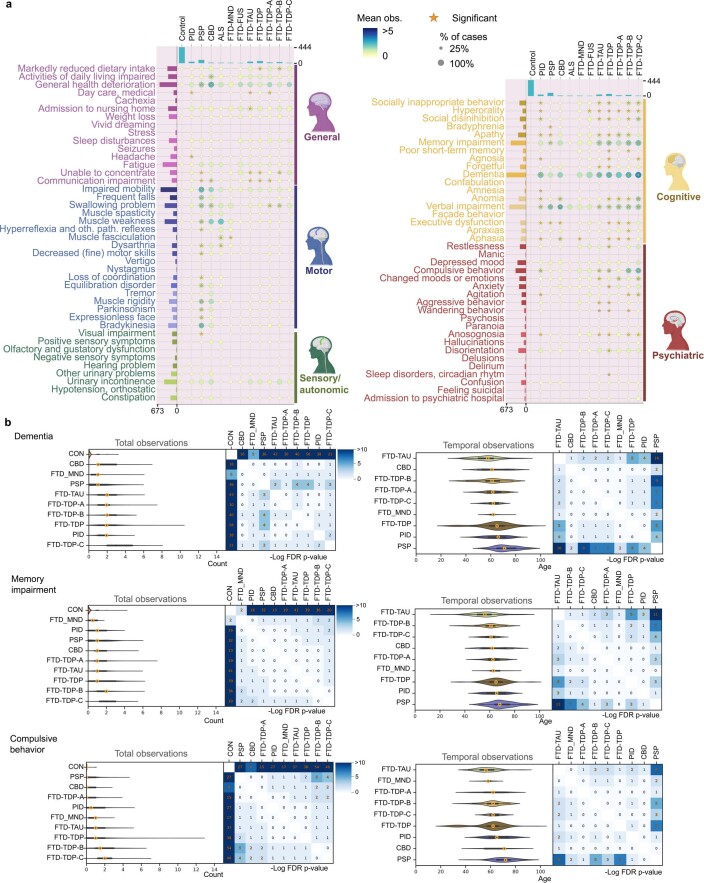
Observational and temporal analysis of FTD subtypes. **a**) Integrated dot and bar plot for FTD subtypes as defined in Fig. 2a. **b**) Violin plots as defined in Extended Data Fig. 3 depicting the observation (n = 708 donors) and temporal distributions of ‘Dementia’ (n = 216 donors)’, Memory Impairment’ (n = 214 donors), and ‘Compulsive Behavior’ (n = 136 donors).

Finally, clinically, it is difficult to differentiate between different FTD subtypes and associated conditions, hence we aimed to identify signs and symptoms that could differentiate subtypes (Extended Data Fig. 4a). ‘Dementia’ observations were significantly lower in PSP cases than in other FTD subtypes, suggesting that this FTD subtype is less affected by dementia, whereas ‘compulsive behavior’ was consistently higher in FTD-TAR DNA-binding protein (TDP)-B, FTD-TDP-C compared with many other FTD subtypes (Extended Data Fig. 4b). Temporally, ‘dementia’ was observed earliest in FTD tauopathy (FTD-TAU) and corticobasal degeneration (CBD) and latest in Pick’s disease (PiD) and PSP. This temporal profile was consistent when these analyses were performed using ‘memory impairment’. Many of these observations were in line with and extended upon earlier work and can contribute toward a better understanding of the relationship between neuropathology and clinical syndromes in FTD disorders^19^.

### Comparing clinical with NDs

**Fig. 3:**
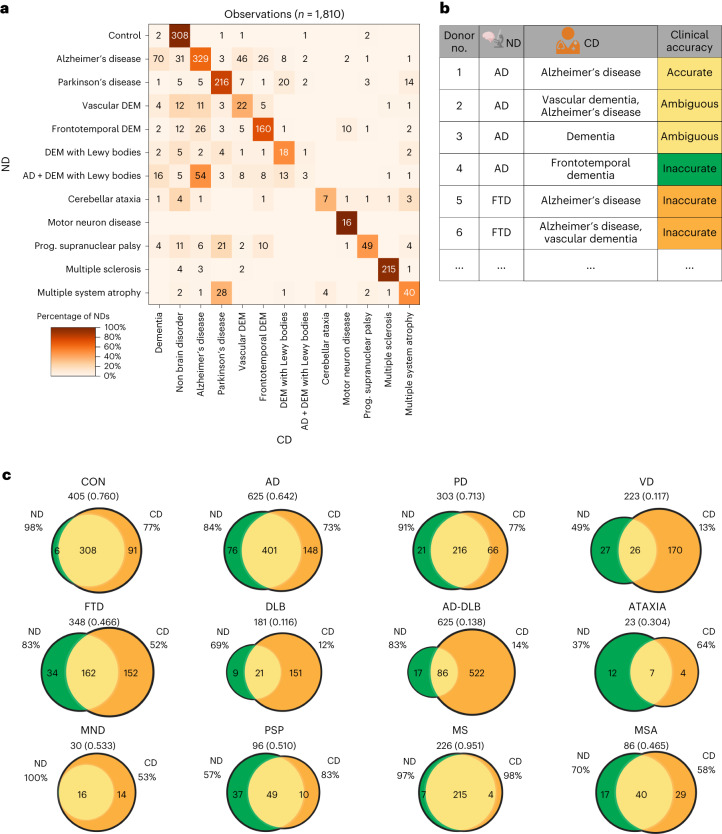
Comparison of CD with ND. **a**, Confusion matrix heatmap of ND (*y* axis) versus CD (*x* axis). Values represent diagnosis observations and hue represents the CD observations divided by the total ND observations for each disorder group. **b**, Table containing illustrative examples of donors to show how CD accuracy was assessed, resulting in three clinical accuracy categories: ‘accurate’, ‘ambiguous’ and ‘inaccurate’. Clinical accuracy is colored to reflect the AD Venn diagram in **c**. **c**, Venn diagrams depicting the intersection of ND and CD for 11 disorders and control cases. Total number of donors and the corresponding JS values are shown below the disorder abbreviation. The percentage represents the proportion of donors with ND who have the same CD (left) and the proportion of donors with CD who have the same ND (right).

As neurodegenerative disorders are frequently clinically misdiagnosed^10,11^, we aimed to determine the diagnostic accuracy of this brain autopsy cohort. For this, we cleaned and linked the CD descriptions to the human disease ontology and compared the resulting CD labels with the ND (Fig. 3a). We then created a set of rules, exemplified in Fig. 3b, to calculate the diagnostic accuracy (Fig. 3c). Most importantly, 84% of neuropathologically defined AD donors and 83% of neuropathologically defined FTD donors were clinically diagnosed as AD (Jaccard score (JS) = 0.642) and FTD (JS = 0.466), respectively. We do note that this also includes ‘ambiguous’ diagnoses, such as the CD dementia. MSA (JS = 0.465) was frequently clinically diagnosed as PD and both VD (JS = 0.117) and PSP (JS = 0.510) were clinically diagnosed as multiple other disorders. Donors with both AD and DLB pathology were most often clinically diagnosed only with AD. These findings suggest that the brain donors of the NBB were also frequently diagnosed inaccurately, in a disease-specific manner.

### Predicting brain disorders using clinical disease trajectories

**Extended Data Fig. 5:**
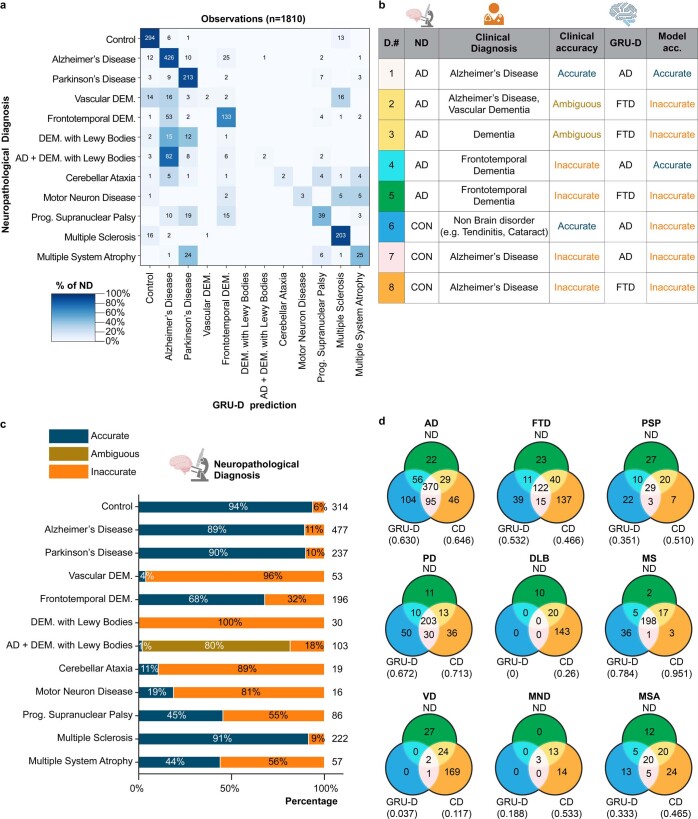
Predictive modeling performance and comparison to clinical diagnosis. Predictive modeling of 1810 brain disorder donors from clinical signs and symptoms. **a**) Heatmap depicting a confusion matrix of Neuropathological Diagnosis (Y-axis) versus GRU-D predicted diagnosis (X-axis). Values represent the number of donors, and the hue represents the percentage of donors in a category compared to the total number of donors with a Neuropathological Diagnosis. **b**) Table with illustrative donor (D.#) examples of Neuropathological Diagnosis (ND), Clinical Diagnosis (CD), and implementation of accuracy parsing rules. **c**) Overview of GRU-D diagnosis prediction accuracy, calculated as percentage of the Neuropathological Diagnosis. **d**) Venn diagrams summarizing the relationship between the Neuropathological Diagnosis (ND), the Clinical Diagnosis (CD) and GRU-D diagnosis prediction (GRU-D), with Jaccard scores between parenthesis.

With the integration of machine-learning models into healthcare practices, we aimed to assess whether the ND could reliably be predicted from clinical disease trajectories. For this, we established a workflow to train a gated recurrent unit (GRU-D) that is particularly developed to work with time-series data with missing values. This model could reliably diagnose most disorders for which we had a higher number of donors (Extended Data Fig. 5a). We also calculated the percentage of accurate diagnoses (in which the ND is considered to be the ground truth) for the GRU-D model (Extended Data Fig. 5b,c) and the CD. Out of 1,810 donors, 1,342 were accurately diagnosed by the model, 83 were ambiguously diagnosed (for example, an AD diagnosis for an AD-DLB donor) and 385 were inaccurately diagnosed. Clinically, 1,236 donors had an accurate diagnosis, 311 were ambiguous (for example, both AD and FTD written down for an AD donor) and 263 were inaccurate. This suggests that the model had a higher percentage of accurate and inaccurate diagnoses simultaneously, owing to the smaller percentage of ambiguous diagnosis.

Compared with the CD, the GRU-D predictions (Extended Data Fig. 5d) performed better for FTD, similarly for AD and PD and worse for MS and PSP. Both model and CD performed equally poorly on DLB, VD, MND and MSA. The GRU-D model performed best for the diagnosis of donors for whom we had at least 100 training cases, whereas most rare cases were missed. Of note, a subset of donors was consistently inaccurately diagnosed by clinicians and the model, indicating that these donors exhibited atypical disease-specific symptoms. We hypothesized that there might be commonalities in the symptomatology of donors with an inaccurate CD and included these inaccurately diagnosed donors as a separate category in the next analysis.

### Dimensionality reduction to characterize the clinical heterogeneity

**Fig. 4:**
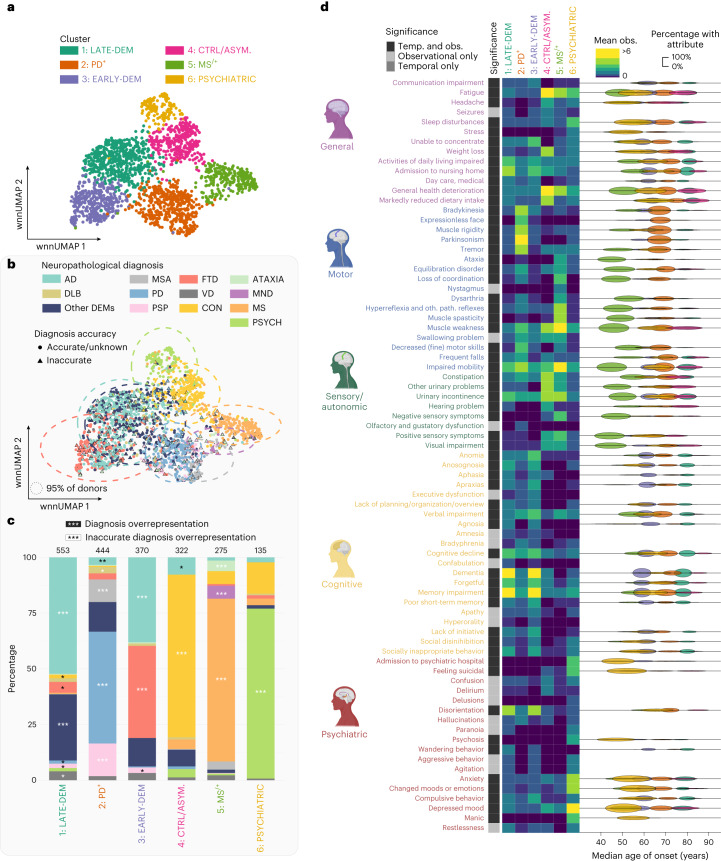
Characterizing clinical heterogeneity through dimensionality reduction. **a**, A wnn-UMAP scatterplot depicting the results of dimensionality reduction and clustering of clinical disease trajectories (*n* = 2,109 NBB donors) based on attribute observations and their temporal manifestation. **b**, A wnn-UMAP scatterplot from **a** depicting the NDs (as colors) and CD accuracy (shape in which circle = accurate or unknown and triangle = inaccurate). **c**, Bar graph showing ND distribution with results of significance testing (one-sided Fisher’s exact test) for overrepresentation of (1) ND across clusters (white asterisk) and (2) inaccurate CDs (black asterisk). FDR-corrected *P* values. ^*^P ≤ 5.00 × 10^−2^, ^**^P ≤ 5.00 × 10^−4^, ^***^P ≤ 5.00 × 10^−6^. **d**, Heatmap showing average number of observations (obs.) of significant attributes (left) and temporal (Temp.) plot showing the median age of onset of significant attributes (right) (two-sided Wilcoxon’s rank-sum test), with width set to the s.d. and height set to percentage of donors experiencing the attribute.

To better understand the clinical heterogeneity of the various brain disorders, we performed dimensionality reduction and clustering on the temporal clinical disease trajectories. Six main clusters were identified (Fig. 4a) that were enriched for: (1) different types of dementias, occurring later in life (LATE-DEM); (2) PD and related disorders that manifest extrapyramidal signs (PD^+^); (3) different types of dementias, occurring at an early age (EARLY-DEM); (4) CON donors and asymptomatic/mild brain disorders (CTRL/ASYM.); (5) motor disorders including MS, MND and ATAXIA (MS^/+^); and (6) psychiatric disorders (PSYCHIATRIC) (Fig. 4b,c). Of note, some disorders were clinically more homogeneous than others. For example, donors with AD, MSA, PD, FTD, MND, MS, PSYCH and CON tend to cluster relatively closely together, whereas donors with VD, PSP and DLB were much more heterogeneous (Fig. 4b).

To obtain insight into the signs and symptoms that differentiate the clusters, we performed a differential analysis (Fig. 4d and Supplementary Tables 7–16). Three distinct observations were made. First, EARLY-DEM and LATE-DEM shared many signs and symptoms, but differed in their temporal manifestation, hence their names. Second, we observed a high number of motor domain attributes in both cluster PD^+^ and MS^/+^, with the PD^+^ cluster having mainly extrapyramidal symptoms and the MS^/+^ cluster mainly ‘muscle weakness’ and ‘impaired mobility’. Third, the PSYCHIATRIC cluster manifested more psychiatric symptoms. These observations largely align with our previous characterizations when we compiled donors according to their diagnosis but, in addition, also illustrate the heterogeneity of these disorders.

In addition, we performed an overrepresentation analysis to determine whether clinically inaccurately diagnosed donors were overrepresented in specific clusters (Fig. 4b,c and Supplementary Table 6). It is interesting that inaccurate FTD, AD, PD, PSP and CON donors were overrepresented in clusters other than their accurately diagnosed counterparts, suggesting that these atypical donors share clinical features with each other that masquerade as another group of disorders. For example, inaccurate AD donors often masquerade as PD^+^ disorders, and vice versa, whereas inaccurate MSA donors often manifest as early or late dementia. This insight elucidates the difficulty of achieving precise diagnoses in a substantial proportion of patients with neurodegeneration.

To assess the validity of the identified clusters, we aimed to perform an enrichment analysis for the *APOE4/4* genotype, which is associated with early AD and more severe neurodegeneration in general^20–23^. Notably, the EARLY-DEM cluster exhibited a robust and highly significant enrichment for the *APOE4/4* genotype (*P* = 5.50 × 10^−8^), the LATE-DEM cluster showed a modest significant enrichment (*P* = 1.32 × 10^−3^), whereas the CTRL/ASYM cluster was significantly underrepresented (*P* = 2.87 × 10^−4^). The remaining clusters did not display significant over- or underrepresentation. These findings offer orthogonal genetic evidence for the validity of these clusters.

### Subclustering analysis to identify data-driven clinical subtypes

To better understand the heterogeneity of donors within a cluster and to identify data-driven clinical subtypes of disease, we performed a subclustering analysis on donors grouped together in a main cluster.

**Fig. 5:**
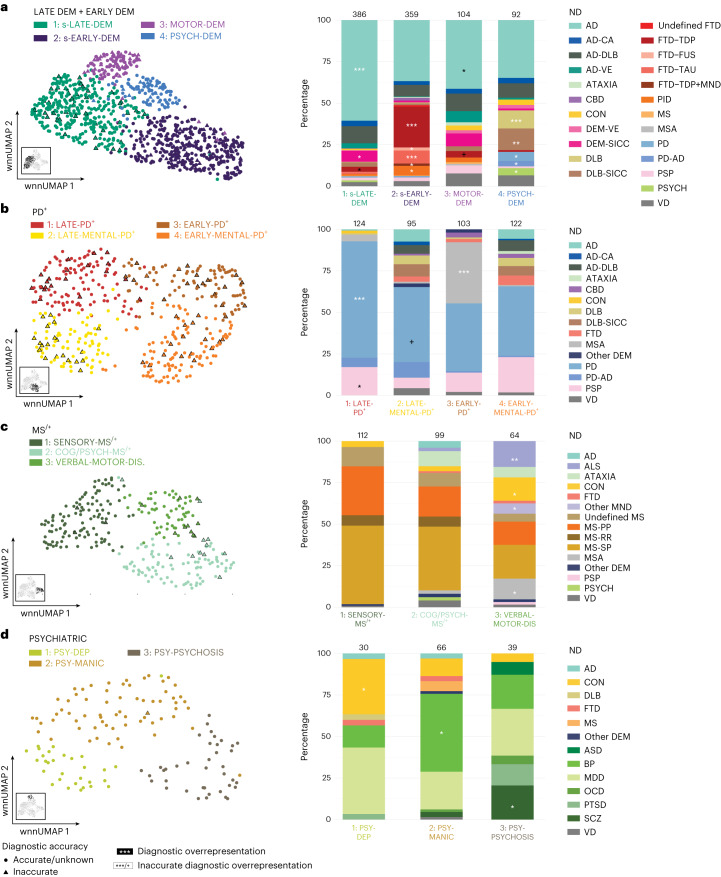
Identification of clinical subtypes. **a**–**d**, Subclustering analysis of 997 EARLY-DEM + LATE-DEM donors (**a**), 444 PD^+^ donors (**b**), 275 MS^/+^ donors (**c**) and 135 PSYCHIATRIC donors (**d**), based on both attribute observations and their temporal manifestation. PP, primary progressive; RR, relapsing–remitting; SP, secondary progressive. Left, wnn-UMAP scatterplot depicting the results of dimensionality reduction and clustering of clinical disease trajectories and CD accuracy (shape in which circle = accurate or unknown and triangle = inaccurate). Right, bar graph showing ND distribution with results of significance testing (one-sided Fisher’s exact test) for overrepresentation of: (1) ND across clusters (white asterisk) and (2) inaccurate diagnoses (black asterisk). FDR-corrected *P* values. ^+^*P* ≤ 1 × 10^−1^, ^*^P < 5.00 × 10^−2^, ^**^P < 5.00 × 10^−4^, ^***^P ≤ 5.00 × 10^−6^.

**Extended Data Fig. 6:**
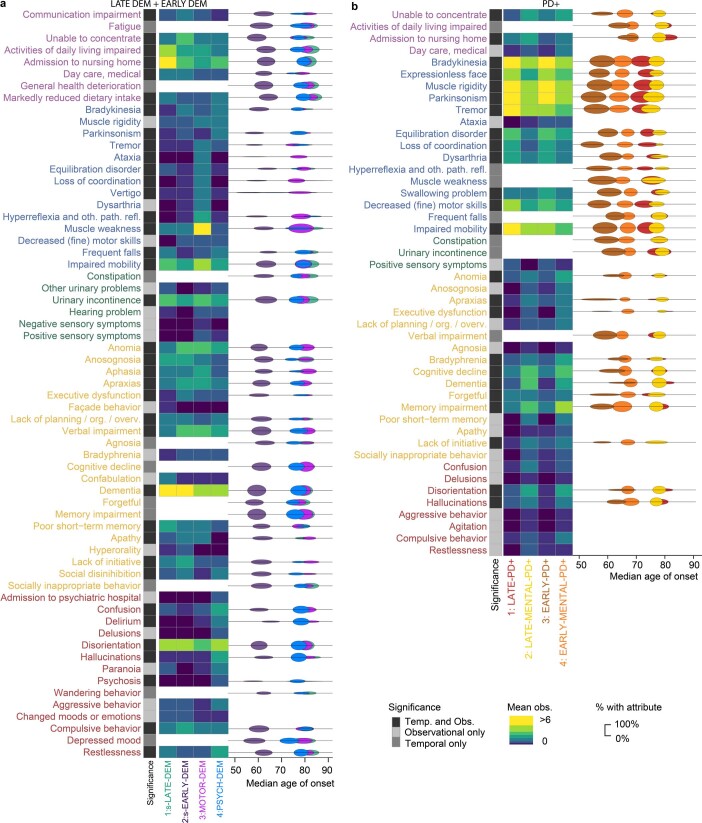
Overview of attributes significant for subclustering analysis of dementias and PD+. Significant attributes for the subclustering analysis of **a**) LATE DEM + EARLY DEM donors (as in Fig. 5a) and **b**) PD+ donors (as in Fig. 5b). For each subcluster the average number of observations (Obs.) of significant attributes are depicted (heatmap, left), as well as a dotplot showing the median age of onset of temporally significant attributes (right) (two-sided Wilcoxon rank-sum test), with width set to the standard deviation and height set to percentage of donors experiencing the attribute.

Subclustering analysis of the merged-DEM clusters (EARLY-DEM and LATE-DEM) resulted in four subclusters (1, s-LATE-DEM; 2, EARLY-DEM; 3, MOTOR-DEM; and 4, PSYCH-DEM) (Fig. 5a). Subcluster 1 (s-LATE-DEM) was significantly enriched for AD and DEM-SICC and inaccurately diagnosed FTD-TDP. Subcluster 2 (s-EARLY-DEM) was significantly enriched for FTD-TDP, FTD-fused in sarcoma (FUS), FTD-TAU and PiD. The symptomatology of this cluster in general manifested at a younger age and showed more ‘compulsive behavior’. Subcluster 3 (MOTOR-DEM) was characterized by ‘muscle weakness’, ‘impaired mobility’ and other motor domain symptoms (Extended Data Fig. 6a). This cluster was also significantly enriched for inaccurate AD, which suggests that AD cases with motor disturbances are clinically frequently misdiagnosed. Subcluster 4 (PSYCH-DEM) was overrepresented for DLB, DLB-SICC, PD, PD-AD and psychiatric donors. This analysis indicates that there might be clinical subtypes of dementia that are manifesting beyond the boundaries of the individual diagnosis that encompasses a relatively early type, psychiatric type, motoric type and generic dementia type. The presence of individual psychiatric and motoric symptoms in subsets of dementia cases has been reported previously^7,24,25^. However, to date, no studies have performed an integrative analysis of the combination of these neuropsychiatric signs and symptoms and their temporal manifestation, resulting in data-driven subtypes. These findings suggest that psychiatric and motor symptoms might be indicative of the clinical subtypes of dementia, potentially mediated by different neurological substructures.

Next, we performed subclustering analysis on the PD^+^ cluster which resulted in four subclusters (1: LATE-PD^+^; 2: LATE-MENTAL-PD^+^; 3: EARLY-PD^+^; and 4: EARLY-MENTAL-PD^+^) (Fig. 5b). It is interesting that two subclusters showed a more limited number of signs and symptoms, one of which had an early onset (EARLY-PD^+^, enriched for MSA) and another with late onset (LATE-PD^+^, enriched for PD and inaccurate PSP donors). Conversely, the remaining two subclusters manifested a broader range of signs and symptoms in the cognitive and psychiatric domains (Extended Data Fig. 6b), again with early onset (EARLY-MENTAL-PD^+^) and late onset (LATE-MENTAL-PD^+^). It has previously been described that patients with PD and related disorders can manifest cognitive and psychiatric problems^7,26,27^. This analysis corroborates these findings and suggests that age of onset and whether mental problems are present are independent disease features.

**Extended Data Fig. 7:**
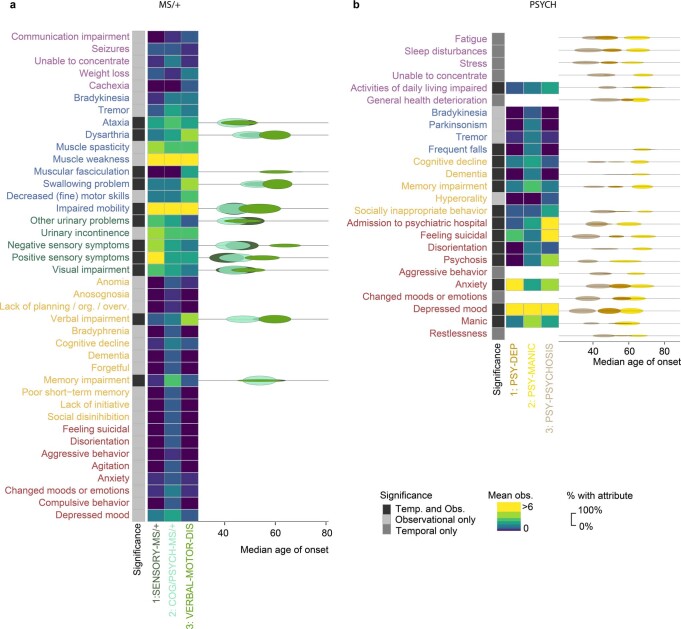
Overview of attributes significant for subclustering analysis of MS/+ and PSYCHIATRIC. Significant attributes for the subclustering analysis of (**a**) MS/+ donors (as in Fig. 5c) and (**b**) PSYCHIATRIC donors (as in Fig. 5d). For each subcluster the average number of observations (Obs.) of significant attributes are depicted (heatmap, left), as well as a dotplot showing the median age of onset of temporally significant attributes (right) (two-sided Wilcoxon rank-sum test), with width set to the standard deviation and height set to percentage of donors experiencing the attribute.

We also performed a subclustering analysis on the MS^/+^ cluster (Fig. 5c) and identified three main clusters: SENSORY-MS^/+^, COG/PSYCH-MS^/+^ and VERBAL-MOTOR-DIS. Most MS donors were clustered in subclusters 1 and 2. The SENSORY-MS^/+^ subcluster manifested fatigue and many other attributes from the sensory/autonomic domain. The COG/PSYCH-MS^/+^ subcluster showed attributes from the cognitive and psychiatric domain. Finally, the third VERBAL-MOTOR-DIS subcluster was significantly enriched for amyotrophic lateral sclerosis and other MNDs, controls and MSA, manifested later in life (Extended Data Fig. 7a). MS, MSA and MND have previously been associated with sensory, mental and motor problems^28,29^. Our analysis expands on these observations and suggests that these motor disorders manifest these symptoms largely independently and these data-driven subtypes are indicative of different neurological substructures being affected.

Increasing lines of evidence suggest that mental illnesses are not discrete categories but that individuals with these disorders manifest behavior along a spectrum of traits^4,30^. Our analysis of the PSYCHIATRIC cluster corroborates this notion because we found three subclusters beyond the confines of the psychiatric diagnosis (Fig. 5d and Extended Data Fig. 7b). Subcluster 1 (PSY-DEP) was enriched for CON and primarily exhibited ‘depressed mood’. Subcluster 2 (PSY-MANIC) was enriched for BP, which was primarily enriched for ‘mania’ and extrapyramidal signs. Subcluster 3 (PSY-PSYCHOSIS) exhibits many observations of ‘psychosis’ and ‘feeling suicidal’, with an early age of onset, and was enriched for SCZ donors.

## Discussion

There is a clear need for new global approaches to study dementia and neurodegenerative disorders^2^. With the advent of machine-learning models, new avenues for improved diagnosis have become feasible. However, publicly available clinical information from a large cohort of neuropathologically defined brain autopsy donors was missing. In the present study, we constructed clinical disease trajectories from medical record summaries from brain donors with various brain disorders. We illustrated the value of this dataset by performing temporal analyses across different dementia subtypes, predictive modeling of end-stage ND and the identification of subtypes of dementia, MS and PD. To better understand, improve diagnostics and develop new interventions and preventive measures for dementia and other brain disorders, we strongly advocate integrative approaches to collect, harmonize and share clinical parameters across brain banks and research institutes. We believe that this is a promising strategy to obtain a much deeper insight into the interindividual factors that contribute to pathophysiological mechanisms. We believe that our strategy to convert textual data to clinical disease trajectories using NLP could function as a road map for other studies.

The clinical trajectories reconstructed in the present study were generated using an NLP model based on medical record summaries, potentially resulting in multiple levels in which misinterpretation or biases could have emerged. First, the retrospectively generated clinical disease trajectories will contain missing values, due to medical doctors not being able to provide all information or not all signs and symptoms being examined during each visit. Fundamentally, this is a typical sampling problem often encountered in different biomedical research fields. We believe that the medical record summaries can be regarded as a sample of the disease manifestation. To deal with missing values, we collapsed the clinical disease trajectories on the year level, imputed additional data points and implemented statistical procedures that were developed to deal with missing data. Second, labeling errors could have been made in the training data and during NLP and might have influenced the results. Other artificial intelligence models, such as generative pretrained transformer-based models and linked entity relationship models (including KRISSBERT) also hold great promise to generate clinical disease trajectories from text data. These unsupervised models might be easier and faster to implement than the supervised approach that we have implemented in the present study. However, the advantage of the supervised models is that the researchers have much more control over the exact definition of the medical term. Third, even though the signs and symptoms used in the present study were identified and defined in several iterations, it is possible that relevant signs and symptoms were not included in the proposed ontology. Fourth, the differential findings concerning the temporal and survival profiles and the clustering between and within NDs might be confounded by additional variables such as medical comorbidities and treatments. Last, the NDs were assigned to donors by different neuropathologists over long periods of time, potentially confounding some of the results.

Neuropathological assessment indicated that a substantial proportion of donors had an inaccurate CD, comparable to previous publications^10,11^. Our work suggests that most of the inaccurate diagnoses were caused by overlapping symptomatology and subsets of atypical donors who manifest consistently differently from the typical disease profile. Misdiagnoses in general not only are harmful to patients because they might not always receive proper medical treatment, but can also majorly confound large-scale studies that rely on CD, such as GWASs and epidemiological studies. Hence, a better understanding of misdiagnoses is critical for both fundamental research and medical care. The diagnostic accuracy of this cohort is also relevant for researchers using these brain tissues. Overall, donors with an inaccurate CD hold potential as a cohort for identifying (bio)markers that could improve the diagnostic process.

Although there is heterogeneity and atypical groups of donors, we theorized that the clinical disease trajectories could serve as a predictor for the ND. We successfully implemented a recurrent neural network to predict the ND for the common diagnoses, although major improvements are still necessary to become clinically relevant. Much larger sample sizes are important, especially for rare and mixed diseases, and we hope that other brain banks will follow our lead.

Finally, the clinical disease trajectories are a representation of the experienced symptomatology. We hypothesized that donors with a shared or similar symptomatology pattern would cluster together in multidimensional space, beyond the confines of specific NDs. These clusters and subclusters offered us insight into disease heterogeneity and symptomatological subtypes of disease. We found that a persistent subset of donors manifest psychiatric symptoms across brain disorders, such as MS, dementia and PD donors with pronounced psychiatric symptoms. This is in line with previous research^27,29,31^ and suggests that different neurological substructures might be differentially affected in these subtypes. Most current postmortem research studies disregard this vital clinical information and implement case–control designs, in which these clinical parameters are neglected. The unique clinical disease trajectories presented in the present study, together with brain autopsy material from the NBB, now allow researchers to study the molecular and cellular features with (clusters of) neuropsychiatric signs and symptoms. We believe that incorporating clinical parameters into brain autopsy material selection and study designs is a critical step toward a more personalized understanding of brain disorders. By capturing the diverse clinical profiles and subtypes of various brain disorders, our research opens the door to future individualized healthcare strategies, where treatment approaches can be customized to each patient.

Taken together, we have established a highly unique resource that could benefit a wide range of researchers, namely: (1) epidemiologists who study the (temporal) symptomatology of various brain disorders, (2) molecular biologists who aim to obtain a deeper understanding of the cellular and molecular features that give rise to neurodegenerative diseases and (3) computational researchers who aim to build predictive models for the diagnosis and prognosis of patients with dementia. These datasets and ontologies are accessible on our website (https://nnd.app.rug.nl).

## Methods

### Netherlands Brain Bank

#### NBB medical record summaries

All adult citizens of the Netherlands can register to become donors in accordance with NBB procedures, which are in full compliance with Dutch and European law. All NBB donors provided informed consent for their tissue and their data to be used for research purposes. The forms and procedures of the NBB were approved by the Free University Medical Center—Medical Ethics Committee (VUmc METC, Amsterdam, the Netherlands). On the death of a donor, the NBB requested in-depth information from the medical specialists and general practitioner/geriatrician about the donor’s specific diagnoses, general health status, surgeries and familial conditions. This information was summarized and translated from Dutch to English by trained medical staff under the auspices of the coordinator medical information, resulting in consistent usage of language and terminology across medical summaries, limiting interdonor and intersummarizer effects.

#### NBB neuropathological examinations and ND

After each brain autopsy, neuropathologists performed extensive macroscopic and microscopic neuropathological examinations for the NBB. The neuropathologists used this information to assign a final diagnostic label to each donor, which we referred to as ‘neuropathological diagnosis’ or ‘ND’ in this paper (Supplementary Table 1). For more information on all NDs used, including their relationship to existing ontogenies (including the *International Classification of Diseases*, 10th revision (ICD-10))^32^, see Table 1 and Supplementary Table 2. We have also established a formal ontology to classify and define all of the implemented NDs that are accessible on our website and BioPortal (https://bioportal.bioontology.org/ontologies/NND_ND). This ND can contain either (1) a clearly defined ND with clinical signs and symptoms such as AD, (2) specific neuropathological traits or NDs that are not associated with a single clinical diagnosis such as hippocampal sclerosis or argyrophilic grain disease (AGD), (3) a psychiatric diagnosis based on clinical observations such as SCZ, (4) specific neuropathologically defined diagnoses that are, or were, final diagnostic labels used exclusively by the NBB, such as DEM-SICC or (5) a neutral label such as ‘control’, indicating the absence of or minimal neuropathological changes and no neurological or psychiatric CD. These ‘control’ donors, however, often suffered from other peripheral diseases, such as cancer. Each donor can have multiple NDs.

### Parsing and matching

#### Parsing of the medical record summaries

The semi-structured medical record summaries were parsed using a broad set of Python-based parsers. Next, the ‘clinical history’ information was parsed per year, and per sentence, setting the stage for temporal profiling through NLP. Sentences without clear year descriptions were categorized as ‘year unknown’. Other time references, such as ‘last 2 months’, ‘last 2 years’ and ‘at birth’, were converted into their respective years. Temporal descriptions spanning multiple years (for example, 2005–2007) were manually transformed into individual years (for example, 2005, 2006 and 2007). Sentences referencing previous years were manually adjusted (for example, ‘in comparison to 2003’).

#### Matching CD to NND—Human Disease Ontology

The values parsed under the header ‘clinical diagnosis’ were manually matched to classes of the Human Disease Ontology (March 2023 release). In some cases, the Human Disease Ontology did not contain all relevant clinical phenotypes (such as primary progressive aphasia and its subtypes, corticobasal syndrome and posterior cortical atrophy), hence we manually modified the ontology to incorporate these labels. The modified Human Disease Otology (NND—Human Disease Ontology) is accessible on our website and BioPortal (https://bioportal.bioontology.org/ontologies/NND_CD). These manually matched CDs were referred to as ‘clinical diagnosis’ or ‘CD’ in the present paper.

### Selection of files from the NBB

#### Selection based on characters

Donors were selected based on sufficient clinical and neuropathological information, defined as the presence of >500 characters in the clinical–neuropathological summaries. The final selection consisted of 3,042 donors, with 199,901 sentences of clinical history data.

#### Selection based on diagnosis

The donors were diagnosed with a wide range of neuropathologically defined brain disorders and received one or multiple NDs, from a list of 89 diagnoses (Table 1 and Supplementary Tables 1 and 2). Donors who were diagnosed with another diagnostic label were excluded. The most common NDs and their numbers, age at death and sex distribution are depicted in Supplementary Fig. 1a.

### Defining signs and symptoms

To identify key signs and symptoms relevant for crossdisorder brain research, we went through several iterations of identifying attributes and labeling sentences from the clinical history of a predefined random set of donors (Fig. 1a,b). The list of signs and symptoms was composed based on three criteria: (1) medical–scientific relevance, (2) sufficient presence in the ‘clinical history’ and (3) unambiguity with respect to the definition. Clinical signs and symptoms used for the CD from the most common neurodegenerative and psychiatric disorders in the NBB were compiled. In addition, attributes that reflect general wellbeing, health and functioning were added. To maintain clinical relevance, we further refined the list by including only signs and symptoms that had sufficient prevalence in the random set to be clinically meaningful. The NND—Clinical History Ontology is now also accessible via our website and BioPortal (https://bioportal.bioontology.org/ontologies/NND_CH). For a comprehensive overview of all initially considered attributes that were not included, please refer to the miscellaneous section of our ontology. Where possible, we have included the Unified Medical Language System identifier for each sign or symptom, providing a clear reference. Ultimately, 90 signs and symptoms were identified and defined (including inclusion and exclusion criteria and examples) and externally validated by a licensed neurologist, encompassing 14 groupings, including ‘disturbances in mood and behavior’, ‘extrapyramidal symptoms’ and ‘cognitive and memory impairment’ in 5 broad domains: psychiatric, cognitive, motor, sensory/autonomic and general (Fig. 1b).

### Labeling of donor files and interannotator agreement

Training data to refine (referring to training or fine-tuning, depending on the model architecture) supervised NLP models was obtained by labeling individual sentences from a random predefined selection of donors. In total, 293 donor files were selected, corresponding to approximately 10% of the data. Scoring and evaluation were performed by trained medical staff of the NBB under the auspices of the coordinator medical information from the NBB. The final training dataset, containing 18,917 sentences, was labeled for the 90 signs and symptoms by 1 scorer (Supplementary Table 3), resulting in a gold standard that was used as input to refine the NLP models for sentence classification. Then, 1,000 sentences were randomly selected from the training set and scored independently by a second scorer to calculate the interannotator agreement.

### NLP model optimization and comparison

The NLP task at hand is the multilabel classification of the 90 attributes in the previously parsed 199,901 sentences. The labeled sentences were stratified and split for crossfold validation (Supplementary Fig. 2a), to refine different NLP models. The Python library, MultilabelStratifiedKFold^33^, was used to split the data into test (20%) and training and validation (80%) fractions. The data were stratified to evenly distribute the different attribute labels over the test and training and validation sets^34^. The training and validation sets were split further using the same MultilabelStratifiedKFold library for the *k*-fold crossvalidation procedure used during model optimization, with a *k* of 5. To ensure accurate comparisons, the same splits were used for the training and validation of every model.

We compared the performance of multiple NLP classification models, to select the best performing model. The best model was used to predict all sentences. We selected two pretrained BERT^35^ models and one T5 (ref. ^36^) model from HuggingFace: PubMedBERT^37^, pretrained on PubMed abstracts, and Bio_ClinicalBERT^38^, pretrained on electronic health records. The standard version of the T5 model was selected from HuggingFace. All transformer models were then fine-tuned on the training data using Simple Transformers^39^. In addition, two common baseline models were used, a BOW and an SVM. The BOW classifier was implemented within a logistic regression framework on word frequency. For the SVM classifier, the Scikit-learn package linearSVC^40^ was used. For BOW and SVM, the sentences were preprocessed through Stop Word Removal and text vectorization, and were wrapped in the Scikit-learn package OneVsRestClassifier^40^.

As our dataset is imbalanced, we assessed model performance using micro-precision, micro-recall and micro-*F*1-score. Hyperparameter tuning for all models was conducted using Optuna^41^, maximizing the average micro-*F*1-score across the 5 crossvalidation folds for 25 trials. Given our emphasis on correct classifications (precision) over identifying every sentence (recall), we first identified the top five iterations of each model type based on the micro-*F*1-score. The final model was then selected based on the highest micro-precision score.

### Descriptive statistics

#### Processing of NLP large language model predictions

The best performing model was used to predict the full corpus of sentences. These predictions were converted into clinical disease trajectories by first grouping the predictions per donor, followed by a conversion into a binary absence/presence matrix of year × attributes. Predictions for which the year was unknown were included in general data exploration but excluded from temporal profiling, modeling or dimensionality reduction.

#### Sign and symptom distribution per main diagnosis

To identify signs and symptoms that were more frequently identified in specific disorders than expected, the total number of signs and symptoms were compiled for all donors with the same ND, and three statistics were calculated and plotted as a dot plot: first, the mean number of observations in sentences for donors belonging to an ND (dot color) and, second, the proportion of donors with a ND that contained minimally one observation of the symptoms (dot size). The color cut-off was set to a maximum of five. The figure also contained a highlighted orange circle around the dot which indicates whether the sign or symptom was of known diagnostic importance for the specific disorder. An asterisk was depicted if the attribute was more commonly observed than expected, given a random background distribution as calculated with a permutation test. The random background distribution was calculated by randomly permuting the diagnosis labels of the individual donor data with 100,000 permutations. The *P* value was calculated as the proportion of observations in which the observed value was higher than the random background, and was multiple testing corrected using the Benjamini–Hochberg false discovery rate (FDR). Moreover, we performed a two-sided *χ*^2^ test to identify whether the significant signs and symptoms (asterisk) per main diagnosis and the signs and symptoms of known diagnostic importance (circles) were overrepresented.

Donors were compiled and studied according to subsets of neuropathological disorders. First, we compiled donors with the most common single ND. Second, we compiled rare and mixed dementias. Last, we compiled different FTD subtypes.

#### Observational profiles of the signs and symptoms

To test whether the number of observations of a given sign or symptom differed between different NDs, we calculated the distribution of the number of year observations per donor within each ND and performed two-sided, pairwise Mann–Whitney *U*-tests using Scipy, followed by an FDR multiple testing correction. These results were visualized as a Seaborn^42^ violin plot which was accompanied by a heatmap showing the results of pairwise significance testing, with −10log(FDR)-corrected *P* values depicted in orange when significant (*P* ≤ 0.01). To account for potential sex bias, we further subsampled the data according to the sex with the lowest numbers to have an equal number of male and female donors for each ND. These subsampled data were also used for the analysis of temporal profiles (see ‘Temporal profiles of the signs and symptoms’) and the survival analysis (see ‘Survival analysis’).

#### Temporal profiles of the signs and symptoms

To test whether the distribution of observations of a given sign or symptom differed temporally between disorders, we performed two-sided, pairwise Mann–Whitney *U*-tests using Scipy, followed by an FDR multiple testing correction. These results were visualized as a Seaborn violin plot as described in ‘Observational profiles of the signs and symptoms’. These results were also plotted as a kernel density plot depicting the distribution of the temporal observations across all donors compiled according to their main diagnosis.

#### Survival analysis

Survival analysis plots depicting the survival of the patients after the first observation of a given sign or symptom were made with Scikit Kaplan–Meier estimator. To test whether the survival after the observations of a given sign or symptom differed temporally between disorders, we performed two-sided, pairwise Mann–Whitney *U*-tests using Scipy, followed by an FDR multiple testing correction. These results were visualized as a Seaborn violin plot as described in ‘Observational profiles of the signs and symptoms’.

### Diagnosis accuracy, predictive modeling and dimensionality reduction

#### Selection of donor files

To select high-quality disease trajectories for predictive modeling and dimensionality reduction, we applied several steps. First, we imputed additional datapoints based on clinically defined rules of thumb. Briefly, signs and symptoms associated with neurodegeneration (column ‘IsNeurodegenerationAssociatedTrait’ in Clinical History Ontology) that were observed in donors suffering from a progressive neurodegenerative disease (column ‘IsProgressiveNeurodegenerativeDisease’ in Neuropathological Diagnosis Ontology) were assumed to remain present after the first observation. Second, for both diagnostic prediction and the analysis of CD, we selected only donors with a single ND including control, AD, PD or PDD, VD, FTD, DLB, ATAXIA, MND, PSP, MS and MSA. We also selected donors with the combination of AD and DLB, the most common form of mixed dementia. For dimensionality reduction, we added donors with a mental illness (MDD, BP, SCZ, post-traumatic stress disorder, autism spectrum disorder, obsessive–compulsive disorder) and donors with other, or mixed, types of dementia (CBD, AD-DLB, AD-CA (congophilic angiopathy), AD-VE, PD-AD, DLB-SICC, DEM-SICC, DEM-SICC-AGD and DEM-VE). Third, for all three analyses, we selected donors for whom the autopsy was performed in or after 1997, as the quality of the summaries improved. Fourth, donors with a diagnosis other than control with fewer than five observations in their clinical disease trajectory were excluded. Together, these criteria resulted in 2,174 donors for dimensionality reduction and 1,810 donors for predictive modeling and the analysis of the CD.

#### Analyzing CD accuracy

Most donors had more than one CD throughout life. To analyze the agreement between CD and ND, we applied the following filtering steps. First, for each ND of AD, PD/PDD, VD, FTD, DLB, AD-DLB, ATAXIA, MND, PSP, MS or MSA, we compiled a dictionary of CDs that is accurate for these 11 disorders based on the modified Human Disease Ontology. Second, we assigned clinical accuracy labels to each donor, being ‘accurate’, ‘inaccurate’ or ‘ambiguous’, as exemplified in Fig. 3b.

Finally, the agreement with the ND was depicted as a confusion matrix of observations and Venn diagrams, with a Jaccard index. Clinically inaccurate donors were further studied in the dimensionality reduction and clustering approach described below.

#### Predicting main diagnosis from clinical disease trajectories with GRU-D

To predict the main diagnosis from the clinical disease trajectories, we implemented a predictive modeling framework (GRU-D) that was ideally suited to deal with temporal missing data^43,44^. The filtered dataset was split into five folds with each fold containing balanced training, validation and testing sets (Supplementary Fig. 6) using the Scikit-learn package StratifiedKFold^40^. Sex, age at death and age when a sign or symptom was observed were included. We trained and optimized this model using default settings for 50 epochs. The test set was used once to estimate final model performance. A confusion matrix of observations was made to show the (dis)agreement with the ND. Again, we calculated the percentage of accurate, inaccurate and ambiguous donors in each disorder group and showed this as a stacked bar plot.

To compare CD, ND and GRU-D-predicted diagnosis, we expanded the Venn diagrams from ‘Analyzing CD accuracy’ with the GRU-D-predicted diagnosis.

#### Dimensionality reduction and clustering of CD trajectories with Seurat

To identify clinical subtypes of neurodegenerative disorders in an unbiased fashion, we implemented Seurat^45^ and clustered donors according to the similarity of their clinical disease trajectories. To balance qualitative information (whether donors exhibited specific signs/symptoms) and temporal information (the age at which these signs/symptoms manifested), we converted the clinical disease trajectories into two separate matrices: a flattened observation matrix (in which the number of observations per symptom were counted over the whole lifespan of each donor) and a temporal matrix (in which the number of observations per symptom were counted in overlapping age bins, for example, signs and symptoms occurring at age 15–45, 20–50 and 25–55 years). The two matrices were loaded in R and then converted into Seurat Assays. For each Seurat Assay we performed normalization, scaling and principal component analysis on all features using default settings. This was followed by a weighted nearest neighbor (wnn) analysis through the function FindMultiModalNeighbors with default settings. Clusters resulting from the function FindClusters were visualized as a Uniform Manifold Approximation Projection (UMAP) and an accompanying identity bar plot. Donors with an inaccurate CD were visualized with a triangle and an ellipse was drawn around 95% of the donors of each ND. We performed two separate Fisher’s exact tests, to determine whether certain disorders or clinically inaccurately diagnosed donors were overrepresented in specific clusters. The function FindMarkers was used to find significant signs and symptoms for both matrices for each cluster, which were visualized as a temporal dot plot. Finally, to investigate differences within clusters that are symptomatologically similar, we performed a subcluster analysis on multiple main clusters.

#### Homozygous *APOE4* genotype overrepresentations

To validate the identified clusters, we collected *APOE* genotype information from donors of the NBB and determined whether homozygous *APOE4* donors were over- or underrepresented across clusters using Fisher’s exact test.

### Reporting summary

Further information on research design is available in the Nature Portfolio Reporting Summary linked to this article.

## Online content

Any methods, additional references, Nature Portfolio reporting summaries, source data, extended data, supplementary information, acknowledgements, peer review information; details of author contributions and competing interests; and statements of data and code availability are available at 10.1038/s41591-024-02843-9.

### Supplementary information

Supplementary Information Affiliations and Supplementary Methods.

Reporting Summary

Supplementary Tables Supplementary Tables 1–16.

## Figures and Tables

**Extended Data Table 1:**
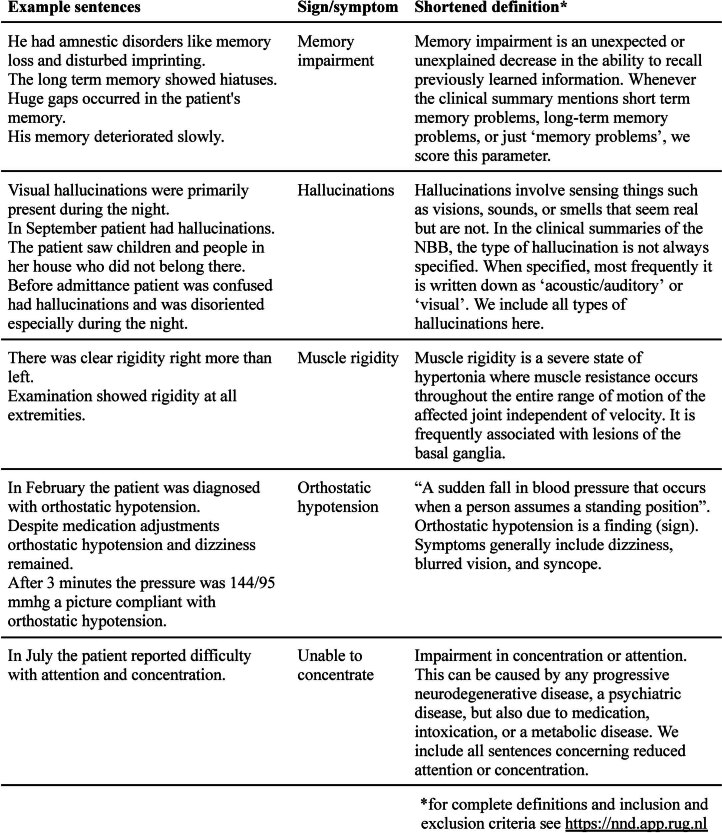
Labeled example sentences

Example sentences that describe the following signs or symptoms and their shortened definition and inclusion/exclusion criteria: ‘memory impairment’, ‘hallucinations’, ‘muscle rigidity’, ‘orthostatic hypotension’ and ‘unable to concentrate’.

**Extended Data Table 2:**
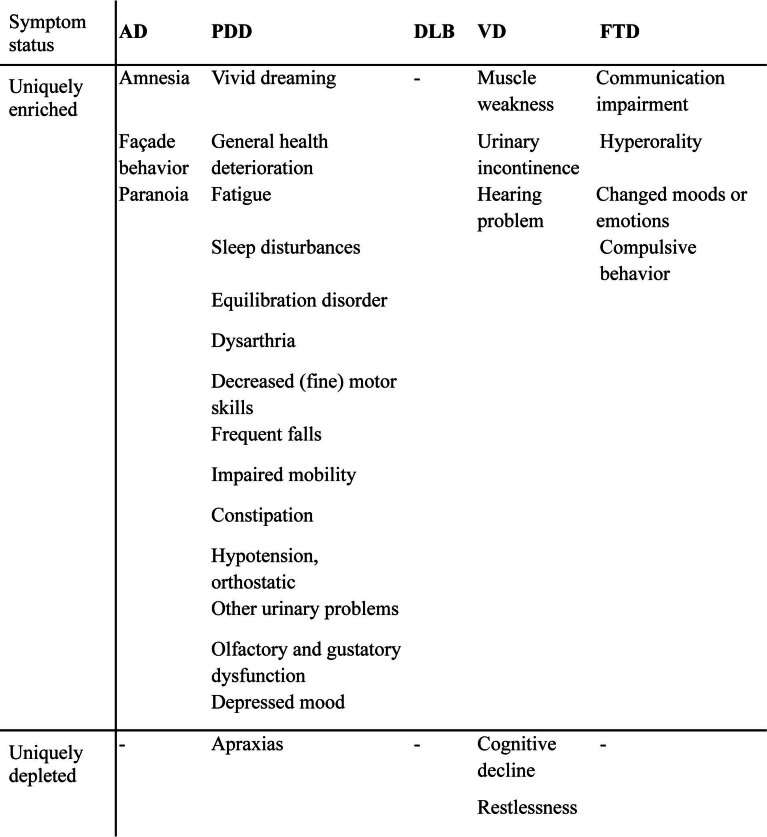
Common dementia symptomatology

Core findings in the group of most common NBB dementias (AD, DLB, PDD, VD and FTD). Overview of signs and symptoms that are significantly enriched in only one disorder (top) and overview of signs and symptoms that are not significantly enriched in one disorder (bottom). An attribute was significant when it was more commonly observed than expected given a random background distribution. The random background distribution was calculated by randomly permuting the diagnosis labels of the individual donor data with 100,000 permutations. The *P* value was calculated as the proportion of observations in which the observed value was higher than the random background and was multiple testing corrected using the Benjamini–Hochberg FDR.

**Extended Data Table 3:**
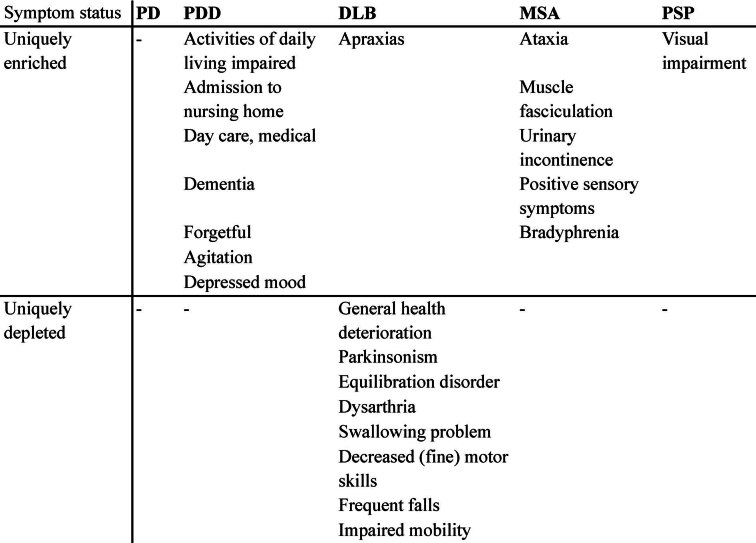
Frequently misdiagnosed disorder symptomatology

Core findings in the frequently misdiagnosed group of disorders PD, PDD, DLB, MSA and PSP. Overview of signs and symptoms that are significantly enriched in only one disorder (top) and overview of signs and symptoms that are not significantly enriched in one disorder (bottom). An attribute was significant when it was more commonly observed than expected given a random background distribution. The random background distribution was calculated by randomly permuting the diagnosis labels of the individual donor data with 100,000 permutations. The *P* value was calculated as the proportion of observations in which the observed value was higher than the random background and was multiple testing corrected using the Benjamini–Hochberg FDR.

## Data Availability

The donor general information, information about the NDs used, the training dataset with labeled sentences and the clinical disease trajectories are included as Supplementary Tables 1, 2, 3 and 4, respectively. We utilized random donor identifiers that do not contain the year of death, in contrast to the NBB identifiers. The age of death information has been adjusted to a 5-year interval. Donors aged >95 years were grouped into the 95+-year category. Donors aged <36 years were grouped into the 35-year category. NDs with fewer than ten donors were added to the parent in the ontology. In addition, all of the unique datasets and supporting ontologies are accessible on our website (https://nnd.app.rug.nl) and supplementary data are available on https://zenodo.org/records/10534111. All supporting ontologies are also publicly accessible on BioPortal. The March 2023 release of the Human Disease Ontology used in the present study can be found on https://github.com/DiseaseOntology/HumanDiseaseOntology/releases/tag/v2023-03-31. The original data and medical record summaries are available from the NBB, but restrictions apply to the availability of these data, which were used under license for the present study, and are not publicly available. However, any researcher can make a data or tissue request to the NBB, by contacting eNBB@nin.knaw.nl. In addition, I. Huitinga is the director of the NBB and can be contacted on http://i.huitinga@nin.knaw.nl to discuss the original NBB data.

## Code availability

Python (v.3.8.2) was used throughout this project in combination with the following software packages: Pandas (v.1.3.5), Fuzzywyzzy (v.0.18.0), Optuna (v.3.0.3) and Simpletransformers (v.0.63.9). Multiple functions from the package Scikit-learn (v.1.0.2) were used to create the BOW and SVM models, such as OneVsRestClassifier, LogisticRegression, LinearSVC and TfidfVectorizer. Seaborn (v.0.12.0) and Matplotlib (v.3.6.0) were used for visualizing data analyses. SciPy (v.1.8.1) and statsmodels (v.0.13.2) were used for statistical analyses. R (v.3.4.4) was used together with Seurat (v.0.12.0) for dimensionality reduction and clustering of clinical disease trajectories. Models used in the present study were fine-tuned versions of the pretrained models PubMedBERT (https://huggingface.co/microsoft/BiomedNLP-BiomedBERT-base-uncased-abstract-fulltext), Bio_ClinicalBERT (https://huggingface.co/emilyalsentzer/Bio_ClinicalBERT) and T5 (https://huggingface.co/t5-base). The fine-tuned NLP model generated during the present study is available from https://huggingface.co/NND-project/Clinical_History_Mekkes_PubmedBert. The trained GRU-D model is available on https://huggingface.co/NND-project/Clinical_History_Mekkes_GruD. Code used for data analysis and model training has now been made publicly available in the following repository: https://github.com/NetherlandsNeurogeneticsDatabase/Clinical_History_NLP.
