# Supplementary material for: Identification of clinical disease trajectories in neurodegenerative disorders with natural language processing
Source: Nat Med. 2024 Mar 12;30(4):1143–53. doi: 10.1038/s41591-024-02843-9 (PMC11031398; doi:10.1038/s41591-024-02843-9)
Supplement: Supplementary file 1 — Affiliations and Supplementary Methods. [file 41591_2024_2843_MOESM1_ESM.pdf]

nature medicine

Article

https://doi.org/10.1038/s41591-024-02843-9

Identification of clinical disease trajectories in neurodegenerative disorders with natural language processing

In the format provided by the authors and unedited

SUPPLEMENTARY INFORMATION

AFFILIATIONS NETHERLANDS NEUROGENETICS DATABASE ADVISORY BOARD

Jörg Hamann[3], Erik W.G.M. Boddeke[1], Morris Swertz[5]

[1]Dept. of Biomedical Sciences of Cells and Systems, Section Molecular Neurobiology, University of Groningen, University Medical Center Groningen, Groningen, The Netherlands
[3]The Netherlands Brain Bank, Netherlands Institute for Neuroscience, Amsterdam, The Netherlands
[5]Dept. of Genetics, University Medical Center Groningen, Groningen, The Netherlands

SUPPLEMENTARY METHODS

This Supplementary Method section expounds further on additional details that may be of interest to researchers seeking an in-depth understanding.

Parsing of the medical record summaries

This section adds further clarification on the Python based parsers used to parse the semi-structured medical record summaries. (Method section 2.2.1 of main manuscript). These parsers first identified specific headers and their synonyms, and secondly captured all pertinent text associated with each header. To accommodate spelling variations, we used fuzz_token_sort_ratio from the FuzzyWuzzy library[1], where a match was determined to be positive when more than 95% of characters were matched with a reference list of all headers. To facilitate this matching process, words in the header sentence were alphabetically arranged, converted to lowercase, and punctuation was removed. The key headers that we aimed to identify in this study were 'clinical history', 'clinical diagnosis', and 'general information'. For some medical record summaries one or more of these headers were not identified. These files were inspected, and reformatted by manually adding the appropriate header.

Signs and symptoms distribution per main diagnosis

This section offers additional detail to the specifics of the permutation test which tests whether an attribute was more commonly observed than expected given a random background distribution (Method section 2.7.2 of the main manuscript). The background consisted of an equal number of donors, who were randomly selected from all the donors belonging to main-diagnosis-categories, from which donors with the diagnosis of interest were removed.

Method section 2.7.2 of the main manuscript also describes selecting different FTD subtypes. This category includes Pick's Disease (PID), FTD-TDP-A (in our cohort associated with progranulin mutations), FTD-TDP-B (association with C9ORF72 mutation), FTD-TDP-C (characterized by predominantly long dystrophic neurites with rare neuronal cytoplasmic inclusions)[2], and associated conditions including Amyotrophic Lateral Sclerosis (ALS), Corticobasal Degeneration (CBD), and Progressive Supranuclear Palsy (PSP).

Analyzing Clinical Diagnosis accuracy

Method section 2.2.2 describes how Clinical Diagnosis accuracy was determined. This extended version offers a more detailed view to aid in reproducibility. To analyze the

agreement between Clinical and Neuropathological Diagnosis we applied the following filtering steps. First, for each Neuropathological Diagnosis of either AD, PD/PDD, VD, FTD, DLB, AD-DLB, ATAXIA, MND, PSP, MS or MSA we compiled a dictionary of Clinical Diagnoses that are accurate for these 11 disorders based on the modified Human Disease Ontology. To illustrate, for MSA, we allowed both "multiple system atrophy" and "striatonigral degeneration" as an accurate Clinical Diagnosis. Second, this dictionary was then used to simplify the Clinical Diagnoses of each donor into one or multiple of these 11 disorders, or into the label "Non Brain disorder" when no brain disorders were mentioned (e.g. "cataract", or "urinary tract infection"). Third, we assigned clinical accuracy labels to each donor, being either "accurate", "inaccurate", or "ambiguous", as exemplified in Figure 3B. For example, a neuropathologically defined AD donor with a Clinical Diagnosis of Alzheimer's Disease, was seen as "accurate", while a neuropathologically defined AD donor with a Clinical Diagnosis of Frontotemporal Dementia was seen as "inaccurate". An "ambiguous" label was assigned to donors with multiple Clinical Diagnoses (e.g. both AD and FTD). The "ambiguous" label was also assigned to AD, VD, FTD, and DLB donors that were diagnosed with the aspecific Clinical Diagnosis label "Dementia". Donors whose Clinical Diagnosis was converted into a "Non Brain disorder" were seen as "accurate" for Control donors.

References

1. CRAN - Package fuzzywuzzyR. Available at: https://cran.r-project.org/web/packages/fuzzywuzzyR/index.html. (Accessed: 23rd August 2022)
2. Mackenzie, Ian RA, et al. "A harmonized classification system for FTLD-TDP pathology." *Acta neuropathologica 122* (2011): 111-113.
