# Supplementary material for: Identification of clinical disease trajectories in neurodegenerative disorders with natural language processing
Source: Nat Med. 2024 Mar 12;30(4):1143–53. doi: 10.1038/s41591-024-02843-9 (PMC11031398; doi:10.1038/s41591-024-02843-9)
Supplement: Supplementary file 2 — Reporting Summary [file 41591_2024_2843_MOESM2_ESM.pdf]

nature portfolio

Corresponding author(s): Nienke J. Mekkes, Inge R. Holtman

Last updated by author(s): 18/01/24

Reporting Summary

Nature Portfolio wishes to improve the reproducibility of the work that we publish. This form provides structure for consistency and transparency in reporting. For further information on Nature Portfolio policies, see our Editorial Policies and the Editorial Policy Checklist.

Statistics

For all statistical analyses, confirm that the following items are present in the figure legend, table legend, main text, or Methods section.

n/a	Confirmed	
☐	☒	The exact sample size (*n*) for each experimental group/condition, given as a discrete number and unit of measurement
☐	☒	A statement on whether measurements were taken from distinct samples or whether the same sample was measured repeatedly
☐	☒	The statistical test(s) used AND whether they are one- or two-sided<br>*Only common tests should be described solely by name; describe more complex techniques in the Methods section.*
☒	☐	A description of all covariates tested
☐	☒	A description of any assumptions or corrections, such as tests of normality and adjustment for multiple comparisons
☐	☒	A full description of the statistical parameters including central tendency (e.g. means) or other basic estimates (e.g. regression coefficient) AND variation (e.g. standard deviation) or associated estimates of uncertainty (e.g. confidence intervals)
☐	☒	For null hypothesis testing, the test statistic (e.g. *F*, *t*, *r*) with confidence intervals, effect sizes, degrees of freedom and *P* value noted<br>*Give P values as exact values whenever suitable.*
☒	☐	For Bayesian analysis, information on the choice of priors and Markov chain Monte Carlo settings
☒	☐	For hierarchical and complex designs, identification of the appropriate level for tests and full reporting of outcomes
☒	☐	Estimates of effect sizes (e.g. Cohen's *d*, Pearson's *r*), indicating how they were calculated

Our web collection on statistics for biologists contains articles on many of the points above.

Software and code

Policy information about availability of computer code

Data collection

Upon death of a donor, the NBB requested in-depth information from the medical specialists and general practitioner/geriatrician regarding the donor's specific diagnoses, general health status, surgeries, and familial conditions. This information was summarized and translated from Dutch to English by trained medical staff. 90 signs and symptoms were identified and defined, and were scored in a subset of medical record summaries from 293 donors. Clinical Diagnoses were matched to Human Disease Ontology diagnoses.

Open source code used for data collection:
Pandas 1.3.5 and Python 3.8.2 were used throughout this project. For the parsing of the medical record summaries Fuzzywuzzy (0.18.0) was used to detect specific headers in the clinic-neuropathological reports.

Data analysis

Data analysis open-source code used:
Pandas 1.3.5 and Python 3.8.2 were used throughout this project.
The Python package MultilabelStratifiedKFold 0.1.7 was used to split the training data.
Multiple functions from the package Scikit-learn (1.0.2) were used to create the BOW and SVM models, such as OneVsRestClassifier, LogisticRegression,LinearSVC, and TfidfVectorizer.

Optuna 3.0.3 was used to optimize the different NLP models.
NLP model performance was analyzed using Scikit-learn classification_report.

Simpletransformers (0.63.9) MultiLabelClassificationModel was used to create the transformer based models. Models used in this study were fine-tuned versions of the pre-trained models PubMedBERT (https://huggingface.co/microsoft/BiomedNLP-BiomedBERT-base-uncased-

abstract-fulltext), Bio_ClinicalBERT (https://huggingface.co/emilyalsentzer/Bio_ClinicalBERT) and T5 (https://huggingface.co/t5-base).

The fine-tuned NLP model generated during the current study is available from https://huggingface.co/NND-project/Clinical_History_Mekkes_PubmedBert.
The trained GRU-D model is available on: https://huggingface.co/NND-project/Clinical_History_Mekkes_GruD.

Code used for data analysis and model training has now been made publicly available in the following repository: https://github.com/NetherlandsNeurogeneticsDatabase/Clinical_History_NLP.

R (3.4.4) was used together with Seurat (0.12.0) for dimensionality reduction and clustering of clinical disease trajectories.
Seaborn (0.12.0) and Matplotlib (3.6.0) were used for visualizing data analyses.
SciPy (1.8.1) and statsmodels (0.13.2) were used for statistical analyses.
No commercial code was used.

For manuscripts utilizing custom algorithms or software that are central to the research but not yet described in published literature, software must be made available to editors and reviewers. We strongly encourage code deposition in a community repository (e.g. GitHub). See the Nature Portfolio guidelines for submitting code & software for further information.

Data

Policy information about availability of data

All manuscripts must include a data availability statement. This statement should provide the following information, where applicable:

- Accession codes, unique identifiers, or web links for publicly available datasets
- A description of any restrictions on data availability
- For clinical datasets or third party data, please ensure that the statement adheres to our policy

The donor general information, the training dataset with sentences and labels, and the clinical disease trajectories are included as Supplemental Tables 1, 15 and 3 respectively. In addition, all of the unique datasets and supporting ontologies are accessible on our website (https://nnd.app.rug.nl). The data can also be found on https://zenodo.org/doi/10.5281/zenodo.10526890. The original medical record summaries that support the findings of this study are available from the Netherlands Brain Bank but restrictions apply to the availability of these data, which were used under license for the current study, and are not publicly available.

Human research participants

Policy information about studies involving human research participants and Sex and Gender in Research.

Reporting on sex and gender

The primary aim of this study was to convert the medical record summaries into standardized disease trajectories, based on 90 cross-disorder sign and symptoms. We did not identify any signs or symptoms for which we a priori assumed that they would be relevant for one sex and or gender only. Nor did we observe striking sex differences in the manifestation of the signs and symptoms.

The second aim of this study was, to interpret the validity of the temporal disease trajectories by interpreting them into the context of different subsets of brain disorders including alpha-synucleopathies, frontotemporal dementias, motor disorders, dementias, psychiatric disorders. As this is a large binary temporal dataset from donors with a broad range of brain disorders (and combinations thereof), we don't suggest that our analyses are in any way exhaustive, and we have not focused on differences between sexes within disorders.

We corrected for sex in our statistical designs for profiling of the manifestation, temporal profiling and or survival analysis of specific signs and symptoms across a subset of disorders. To this end we used a subsampling approach where equal numbers of male and female donors were analysed.

The most common Neuropathological Diagnoses and their numbers, age at death, and assigned sex distributions were depicted (and depicted in Suppl. Fig. 1A). Importantly, we supply sex and gender data for other researchers to study this in more detail for specific disorders.

Population characteristics

This study covers 3,042 brain donors that were processed between 1982 and 2020. 1,695 were female, 1,347 were male. The average age was 74.61 (+/- 13.45). Donors could no clinical or neuropathological indication of brain disorder(n=445), Alzheimer's Disease (n=720), Frontotemporal Dementia (n=220), Multiple Sclerosis (n=259), Parkinson's Disease (n=134), or one of the many other brain disorders described in the manuscript. For an overview of all diagnoses, please see the Supplementary Tables.

Recruitment

All adult citizens of the Netherlands can register to become donors in accordance with NBB procedures which are in full compliance with Dutch and European law. All NBB donors provided informed consent for their tissue and their data to be used for research purposes.
We have identified the following potential sources for selection bias:

- We anticipate that there is an education bias within the NBB cohort, with a higher average education level of the NBB donors when compared to the general population. This could impact the results since a higher education level is correlated with a higher life expectancy and lower rates of cognitive decline.
- We also anticipate that individuals in our cohort suffer from brain diseases more frequently than expected, as patients suffering from brain diseases tend to be more willing to participate in fundamental research than individuals without a history of brain pathology.
- This brain autopsy cohort almost solely consists of individuals with Dutch/Caucasian background, potentially limiting generalizability to other ancestries.

Ethics oversight

The forms and procedures of the NBB were approved by the Free University Medical Center Medical Ethics Committee (VUmc METC, Amsterdam, the Netherlands). This study protocol did not warrant any ethics oversight.

Note that full information on the approval of the study protocol must also be provided in the manuscript.

Field-specific reporting

Please select the one below that is the best fit for your research. If you are not sure, read the appropriate sections before making your selection.

☒ Life sciences ☐ Behavioural & social sciences ☐ Ecological, evolutionary & environmental sciences

For a reference copy of the document with all sections, see nature.com/documents/nr-reporting-summary-flat.pdf

Life sciences study design

All studies must disclose on these points even when the disclosure is negative.

Sample size

3,042 Donors were used in the final analyses. Sample size was not predetermined.

Brain autopsies are a highly specialized, time-consuming, and staff-intensive procedures, with many practical limitations (including limited donor registrations). Hence, we did not do any sample size predetermination, as it was unfeasible to increase the number of brain autopsies for specific disorders within the confines of this study. Moreover, before the study started it was impossible for us to determine how many signs and symptoms would be described in the average medical record summaries and how those would differ between disorders. The number of donors varies per neuropathological diagnosis, with some diagnosis being highly frequent (such as AD, PD, MS). Many other disorders are very rare, with only one or 2 donors in our autopsy cohort. We have restricted most of our analyses to neuropathological diagnosis with larger sets (minimal N > 8), with these numbers we are able to pick up on medically relevant information

Data exclusions

Donors were selected based on sufficient clinical and neuropathological information, defined as the presence of more than 500 characters in the clinical-neuropathological summaries. Donors under the age of 21 were also excluded from the analysis, as there were only a few, and this small subset was much younger than all other donors, making it difficult to use this group as a ‘control’ group.

Replication

When comparing different NLP model architectures, we used the same sets of training data to assess how well each model performed. The results could theoretically be seen as replicates. We tested 5 model architectures, and each architecture was optimized in 30 trials, meaning we have 150 replicates. The best performing model was chosen to predict the full corpus of text, meaning that our downstream analyses are based on an independent experiment.

Randomization

We randomly selected a subset of donors for whom individual sentences from medical record summaries were labeled to generate training data. Additionally, we randomized the sentences that we used in our model k-fold cross validation approach. Finally, we randomized the donors that were used in the validation of the Neuropathological Diagnosis prediction model.

Blinding

For each sentence, in the labeled dataset, the signs and symptoms that were positively stated were scored blind, without knowledge of donor medical background or neuropathological status.

Reporting for specific materials, systems and methods

We require information from authors about some types of materials, experimental systems and methods used in many studies. Here, indicate whether each material, system or method listed is relevant to your study. If you are not sure if a list item applies to your research, read the appropriate section before selecting a response.

Materials & experimental systems

n/a	Involved in the study
☒	☐ Antibodies
☒	☐ Eukaryotic cell lines
☒	☐ Palaeontology and archaeology
☒	☐ Animals and other organisms
☒	☐ Clinical data
☒	☐ Dual use research of concern

Methods

n/a	Involved in the study
☒	☐ ChIP-seq
☒	☐ Flow cytometry
☒	☐ MRI-based neuroimaging
